# Small molecule activators of the Trk receptors for neuroprotection

**DOI:** 10.1186/1471-2202-9-S2-S1

**Published:** 2008-12-03

**Authors:** Nicholas JG Webster, Michael C Pirrung

**Affiliations:** 1 Veterans Medical Research Foundation and VA San Diego Healthcare System, San Diego, California 92161, USA; 2 Department of Medicine, University of California, San Diego, La Jolla, California 92093, USA; 3 Department of Chemistry, University of California, Riverside, California 92521, USA

## Abstract

The neurotophin signaling network is critical to the development and survival of many neuronal populations. Especially sensitive to imbalances in the neurotrophin system, cholinergic neurons in the basal forebrain are progressively lost in Alzheimer's disease. Therapeutic use of neurotrophins to prevent this loss is hampered, however, by a number of pharmacological challenges. These include a lack of transport across the blood-brain barrier, rapid degradation in the circulation, and difficulty in production. In this review we discuss the evidence supporting the neurotrophin system's role in preventing neurodegeneration and survey some of the pharmacological strategies being pursued to develop effective therapeutics targeting neurotrophin function.

## Background

### The neurotrophins and their receptors

Nerve growth factor (NGF) was the first neurotrophin discovered and is the prototypical member of a family of structurally related proteins including brain-derived neurotrophic factor (BDNF), neurotrophin (NT)-3, and NT-4/5. Neurotrophins have been identified in many vertebrate lineages, including reptiles, amphibians, fish, birds, and mammals [1]. Not all neurotrophins have been found in all species; NT-4/5 has not been found in chicken and NT-6 and -7 are found only in fish. More distantly related genes have been found in the *Agnatha *species lamprey and hagfish. Phylogenetic analysis indicates that NGF and NT-3 form a subfamily as do BDNF and NT-4/5. The neurotrophins bind two classes of receptor, the common p75NTR, which is a member of the tumor necrosis factor receptor family [2,3], and the tropomyosin-related kinase (Trk) receptors, members of the large tyrosine kinase receptor family [4]. Three Trk receptors have been identified in vertebrates, and more distantly related receptors have been found in lamprey and amphioxus. TrkA and TrkC form a distinct phylogenetic subfamily [1]. All neurotrophins bind p75NTR with similar affinity (K_d_~10^-9 ^M), but binding to the Trk receptors is more selective. NGF binds the TrkA receptor, BDNF and NT-4/5 bind the TrkB receptor, and NT-3 primarily binds the TrkC receptor [5]. These binding specificities are consistent with the phylogenetic separation into distinct subfamilies. NT-6 and -7 are more closely related to NGF than the other neurotrophins and bind TrkA [1]. The common p75NTR also enhances binding of NGF to TrkA receptors to create high affinity binding sites (K_d_~10^-11 ^M), possibly by binding to different surfaces on the NGF dimer [6]. p75NTR can also bind precursor forms of the neurotrophins. For example, pro-NGF does not bind TrkA, but causes cell death by binding to p75NTR [7,8].

These neurotrophins function to support the growth and survival of many neuronal populations [5,9-11]. NGF-deficient mice die shortly after birth and show a decrease in sensory and sympathetic neurons, but basal forebrain cholinergic neurons develop relatively normally. However, this perinatal lethality can be rescued by transgenic expression of NGF under the K14 keratin promoter, which restores the sensory and sympathetic neuronal populations [12]. These rescued mice exhibit reduced cholinergic innervation in the cortex and hippocampus, but this can be restored by intracerebroventricular delivery of NGF [13]. Disruption of a single NGF allele causes deficits in memory acquisition and hippocampal cholinergic innervation, suggesting that NGF is required for the formation and maintenance of correct innervations [14]. BDNF-deficient mice also die shortly after birth due to defects in brain and sensory, but not motor, neuron development [15]. Long-term potentiation and mechanosensation are impaired in heterozygous BDNF knockout animals [16,17]. NT-3 deficient mice show severe movement defects and die shortly after birth with complete absence of spinal proprioceptive afferents [18]. The number of muscle spindles was reduced in heterozygous NT-3 knockout mice. NT-3 deficiency also causes a decrease in the number of sympathetic cervical ganglion neurons, lingual innervations, and sensory neuron precursor cells [19,20].

The three Trk receptors show distinct patterns of expression throughout the mammlian brain and peripheral nervous system [4,21-24]. TrkA is expressed exclusively by cholinergic neurons in the basal forebrain. TrkB and TrkC are highly expressed in the hippocampus. In the peripheral nervous system, these receptors are expressed on overlapping sets of sensory and motor neurons. None of the Trk receptors are essential for embryonic development and knockout mice are born in the expected ratios. However, the mice fail to thrive and die shortly after birth. TrkA-deficient mice show a marked loss of cholinergic neurons in the forebrain and sensory and sympathetic neurons in the trigeminal, superior cervical, and dorsal root ganglia [25-28]. Mice lacking TrkB show decreased synaptogenesis and mossy fiber maturation in the hippocampus, as well as severe sensory deficits in the vestibular and cochlear ganglia [29-31]. TrkC-deficient mice also show decreased hippocampal synaptogenesis and display abnormal movements due to loss of proprioception and muscle afferents [30-32]. Heterozygosity for a *trk *allele permits survival, but structural alterations are apparent in aged mice [14,33,34]. TrkC is also expressed on non-neuronal cells and supports glial development [35]. Multiple alternatively spliced isoforms have been observed for TrkA, TrkB, and TrkC, especially in non-neuronal cells [36-39]. Some of these isoforms lack the cytoplasmic tyrosine kinase domain, but retain selective signaling and may inhibit neurite outgrowth [37,40,41].

Due to their ability to protect multiple neuronal cell types from apoptosis, there is considerable interest in whether neurotrophins can stimulate neuronal regeneration *in vitro *and in model systems [42-45]. If neurotrophins can prevent or reverse neuronal cell loss, they would make good therapeutic targets in neurodegenerative diseases, and in brain or spinal cord injuries [46,47]. One complication, however, is that neurotrophins also bind to p75NTR. Activation of this receptor may cause cell death rather than survival, as p75NTR-/- mice show reductions in neuronal cell death after pilocarpine-induced seizures compared to wild-type [48,49]. Therefore, the final effect of a neurotrophin is a balance between the cell survival signal derived from the Trk receptor family and the cell death signal from p75NTR. Indeed, neurotrophins cause cell death in approximately 30% of cultured hippocampal neurons expressing p75, but which lack the cognate Trk receptor, potentially limiting the therapeutic utility of the neurotrophins themselves [50].

### Neurotrophins, Trks, and neurodegeneration

In Alzheimer's disease (AD), one of the most severely affected systems is the cholinergic neurons projecting from the basal forebrain to the neocortex. There is a strong correlation between the loss of these cholinergic neurons and the loss of memory [51]. Cholinesterase inhibitors can prevent the breakdown of acetylcholine that results in the partial restoration of memory and decreased confusion, but despite this initial improvement, as more cholinergic neurons are lost, there is a continual, gradual loss of function. Many reports have documented the ability of NGF to improve cholinergic function *in vitro *and to prevent lesion-induced degeneration in rodents and primates, as well as age-associated declines in rats and primates [46,50,51]. NGF is expressed in a number of cell types in the brain, including astrocytes, but not in microglia or oligodendrocytes [52]. Correlative studies have found defects in the NGF system in early stages of AD that may indicate a causative role. NGF mRNA levels are not altered in AD. Total NGF protein is relatively normal, but the ratio of proNGF to mature NGF is increased [53,54]. TrkA expression is reduced, but p75NTR amounts are unchanged in AD [55,56]. The reduction in TrkA suggests a relative deficit in NGF signaling, as NGF positively regulates *trkA *gene expression. proNGF may increase apoptotic signaling via p75NTR, altering the balance between neuronal survival and death. The importance of NGF is underscored by studies in a transgenic model of neurodegeneration. Mice expressing blocking antibodies to NGF, specifically in the adult brain, showed reduced cholinergic innervations of the cortex and impairments in synaptic plasticity [57]. Similarly, short-term neonatal administration of anti-NGF antibodies by intracerebroventricular (i.c.v.) injection in mice leads to altered exploratory behavior on a hole board [58]. BDNF may play an important role too. In AD, not only are BDNF mRNA and protein levels decreased in basal forebrain, cholinergic neuron target tissues – for example, cortex and hippocampus [59] – but local levels of BDNF in the nucleus basalis are reduced [60]. Hippocampal BDNF levels are increased by exercise, thought to delay decline in patients with AD [61]. Adenoviral expression of BDNF enhances neuronal plasticity, increases long-term potentiation formation, and improves performance on behavioral tests in a rat model with cognitive deficits [62]. NT-3 levels in the motor cortex are also lower in AD patients than controls [63], but other regions appear unaltered [64,65]. Furthermore, the receptors TrkB and TrkC are decreased in cholinergic basal forebrain neurons in AD [55].

Neurotrophins may also be a promising therapy for neuroprotection in traumatic brain injury. In a study of 14 children with severe traumatic brain injury, NGF levels in the cerebrospinal fluid were normal 2 hours post trauma, but increased 5-fold at 24 hours post trauma [66]. In contrast, BDNF levels were elevated >25-fold 2 hours post trauma, but decreased to 3-fold of normal at 24 hours. Higher NGF levels at 24 hours post-trauma correlated with better clinical outcome. These elevations in neurotrophins have also been seen in rodent models of brain injury and are thought to be a protective mechanism to minimize neuronal loss [67-69]. Kainate-induced injury increases BDNF and NGF levels in the hippocampus of Fisher 344 rats [69]. The increase in BDNF was diminished in aged rats, suggesting reduced spontaneous healing with aging [70,71]. Cortical and hippocampal NT-4/5 levels are elevated in rats subjected to a lateral fluid percussion injury, and NT-4/5 knockout mice showed more extensive Cornu Ammonis (CA) pyramidal cell loss and showed impaired motor recovery compared to wild-type mice Prolonged infusion of NT-4/5 prevented 50% of the pyramidal cell loss. In another study, BDNF and NT-3 were elevated in the rat thalamus following percussive brain injury [68]. Glucocorticoids are often prescribed following brain injury. Administration of dexamethasone, a synthetic glucocorticoid, elevates brain levels of NGF, BDNF, and NT-3 following injury [73-76]. Adrenalectomy prevents the trauma-induced increase in NGF, underscoring the essential role of endogenous glucocorticoids in inducing the protective neurotrophins [77]. NGF infusion promotes local axonal regeneration in spinal cord injury, but does not extend beyond the site of infusion, which led to the suggestion that neurotrophin gradients are required [78,79]. To test this idea in a transgenic model, Pettigrew *et al. *[80] expressed NGF throughout the brain under control of the GFAP promoter. Neurons grafted into the transgenic animals showed extensive parallel axonal regeneration extending beyond the astroglitic scar, demonstrating that gradients are not needed for parallel fiber growth, and that global expression of NGF is beneficial. BDNF is required for sprouting of hippocampal CA3 pyramidal cell axons, and either BDNF or NT-3 supports engraftment of CA3 cells in kainite-lesioned rats [81,82]. NT-4/5 can also increase CA2/3 cell survival following fluid percussion injury, but did not improve performance on behavior tests [83]. The beneficial effects of neurotrophins may not be limited to neuronal survival, as BDNF reduces blood-spinal cord barrier permeability following spinal cord injury, and reduces leakage of serum proteins [84]. BDNF, and its receptor TrkB, are also required for the anti-depressant effect of imipramine and fluoxetine in rodent models, suggesting that neurotrophin therapy may also have beneficial effects in post-traumatic stress and other depressive disorders [85,86].

## Neurotrophin therapy

In preclinical and clinical findings, neurotrophins are thought to be a promising therapy for peripheral neuropathies and neurodegenerative diseases, including AD [87] and Parkinson's disease [88]. However, neurotrophins do not make good drug candidates due to their poor pharmacokinetic behavior and bioavailability at the desired targets. One of the major hurdles for neurotrophin therapy is the lack of passage of peptide hormones across the blood-brain barrier [89,90]. Peripheral administration of peptide hormones only leads to a small increase in their intra-cerebral concentration. This has necessitated complicated methods of delivery, such as via the olfactory neural pathway [91-93], *ex vivo *gene therapy by intracranial injection of NGF-expressing fibroblasts [94], or placement of in-dwelling catheters to allow neurotrophin infusion. Intranasal administration of NGF rescues memory defects in a mouse model of AD [95]. Preliminary trials of i.c.v. infusion of recombinant mNGF for 3 months have shown some benefit in small numbers of AD patients [96]. *Ex vivo *gene therapy using the patients' own fibroblasts infected with a retrovirus to express human NGF is currently being tested in phase I trials, but recent problems associated with gene therapy make it unlikely that such an approach will succeed in the near future [94].

As a result, considerable effort has been devoted to finding neurotrophin peptidomimetics. These are small molecules that mimic the binding of selective peptides and elicit the desired neuroregenerative responses of neurotrophins. Much of this work is driven by the crystal structures of the neurotrophins and their receptors, and structure-function studies of small peptides [97,98]. NGF contacts the TrkA receptor through residues in β-hairpin loops 2 and 4, and residues in the amino- and carboxyl termini. A dimeric, cyclized peptide derived from loop 4 activates TrkA and has NGF-like neurotrophic effects [99]. Other β-turn mimetics have also been developed and optimized for neurotrophic activities *in vitro *[100,101]. NGF contacts p75NTR through residues in loop 1 and peptidomimetics have been developed that block the binding of NGF, preventing apoptotic cell death driven by proNGF activation of p75NTR [102]. Taking a slightly different approach, small peptides have also been developed that prevent the association of the Trk receptors with cellular tyrosine phosphatases. Treatment of PC12 cells with a cell-permeable version of the leukocyte common antigen receptor (a tyrosine phosphatase), expressed in neurons, causes neurite outgrowth in a TrkA-dependent manner, presumably by preventing de-phosphorylation of TrkA [103]. A peptidomimetic, cerebrolysin (N-PEP-12) from Ebewe Pharma, is showing promise in phase II clinical trials [104].

## Small molecule, non-peptide neurotrophic factors

The development of small molecules promoting neurotrophic function has been recently reviewed [105]. These are small molecules that can alter neurotrophin function in a manner independent of ligand binding to receptor. The mechanisms can be varied. Some agents modulate the expression of the neurotrophins themselves, while others act by enhancing neurotrophin action. In the former class, a purine-hypoxanthine derivative, leteprinim (Neotrofin), has been shown to elevate neurotrophin levels (including NGF) in animals, and caused increases in acetylcholinesterase labeling in lesioned rats [106]. In humans with mild-to-moderate AD, this molecule improved cognitive scores in memory, executive function, and attention tests [107]. It also increased metabolic rate especially in the cerebellum, and sensory and prefrontal cortices. Leteprinim showed promise in a phase II clinical trial for the treatment of AD but is now being developed for peripheral neuropathy. Similarly, retinoids, AMPA (alpha-amino-3-hydroxy-5-methyl-4-isoxazolepropionic acid) receptor agonists, selective serotonin reuptake inhibitors, serotonin/norepiephrine reuptake inhibitors, and tricylic antidepressants all increase neurotrophin expression [105].

As for the agents acting by enhancing neurotrophin action, xaliproden is a combined NGF potentiator and serotonin 5-HT_1A _receptor agonist that also reduces chemotherapy-induced peripheral sensory neuropathy but has yet to show efficacy in cognitive decline in AD. Its ability to enhance NGF action *in vitro *is mimicked by tyrosine kinase inhibitors [108]. This is reminiscent of the enhancement of NGF action by inhibitors of mixed-lineage kinases (MLKs), for example, K252a, CEP1347, BMS355249, or L-753,000 [109-112]. For many years, K252a was thought to be a weak TrkA inhibitor, but was subsequently shown to be a potent inhibitor of the MLKs. More specific MLK inhibitors that lack the TrkA inhibitory activity can still enhance neurotrophin action. Nitric oxide donors also contribute to neuronal survival through transactivation of TrkA [113-115]. The protective effect requires cyclic GMP and protein kinase G, but the downstream target is not known. Activation of G-protein coupled receptors by adenosine, or pituitary adenylate cyclase activating peptide (PACAP), also leads to transactivation of TrkA, stimulation of downstream signaling, and neuroprotection [116,117]. These effects may be related to the known redox regulation of TrkA. Antioxidants, such as N-acetylcysteine, blocks NGF-induced neuronal differentiation, whereas buthionine sulfoximine, which reduces cellular glutathione levels, enhances neuronal differentiation [118].

Other studies have focused on small molecule, direct activators of the TrkA receptor. A cell-based chemical genetic screen for compounds that can protect SN56 neuroblastoma cells against apoptosis in a TrkA-dependent manner identified gambogic amide as a selective agonist for TrkA [119]. Gambogic amide is the major active ingredient of gamboge, a traditional Chinese medicine. This compound binds TrkA, but not TrkB or TrkC, and causes receptor dimerization and phosphorylation. Gambogic amide stimulates neurite outgrowth in PC12 cells, reduces neuronal cell death in a kainate-induced seizure model, and reduces infarct volume in a middle cerebral artery occlusion (MCAO) stroke model [119].

Our studies have focused on the asterriquinone class of compounds. The asterriquinones are naturally occurring bis-indolyl-dihydroxyquinones that were originally identified as activators of the insulin receptor [120,121]. The molecules are small and readily cell-permeable, and act directly on the receptor tyrosine kinase domain, although its mechanism of activation is not known. The original compound, demethylasterriquinone-B1 (DAQ-B1), was demonstrated to cross the blood-brain barrier and activate hypothalamic signaling when given orally [122]. Therefore, we hypothesized that similar compounds could potentially activate signaling in the central nervous system and be used as oral NGF activators for neurotrophin therapy. These compounds would have the very important additional advantage of targeting the kinase domain of TrkA and therefore not activating the p75NTR receptor. To facilitate identification of TrkA activators, we developed a combinatorial library of structurally related asterriquinones and screened them against TrkA [123]. Out of 334 asterriquinones screened, we identified 35 that had agonist activity >50% that of NGF. Dose-dependent toxicity was also measured for this library and TrkA activation and toxicity were modeled mathematically using quantitative structure activity relationship (QSAR) models. There was no correlation between activation of TrkA and cellular viability, suggesting that toxicity is not dependent on agonist activity. Based on the library screen, we picked compound 5E5 for further evaluation. This compound is a potent activator of TrkA (200% the activity of NGF) and a partial activator of TrkC (80% the activity of NT-3), but does not appreciably stimulate TrkB or the insulin receptor. Interestingly, this compound has additive effects with NGF and at low doses is able to potentiate the effect of NGF to activate TrkA and downstream signaling. 5E5 enhances neurite outgrowth and neuronal differentiation of PC12 cells in the presence of a low dose of NGF that alone is inefficient at promoting neurite outgrowth.

## Conclusion

Progress continues in the development of strategies for neuroprotection in AD. Both the central role for neurotrophins in mediating neuronal survival, and the positive effects using recombinant NGF or peptidomimetics in small clinical trials, suggest that the neurotrophin system would be a good therapeutic target in AD. Multiple approaches are being pursued, though many have their limitations. Peptidomimetics offer greater specificity, but are less readily delivered. Small molecule neurotrophin mimetics are easily delivered, but less selective, and may have non-Trk-mediated side effects. In the end, molecules potentiating the effect of endogenous neurotrophins are particularly appealing as this may avoid the potential complications of systemic neurotrophin stimulation. In this way, neurotrophin action would only be enhanced where endogenous neurotrophins are found. Many of these studies establish proof-of-principle, and it is hoped that they will spur development of second and third generation therapeutics with better selectivity and potency.

## List of abbreviations used

AD: Alzheimer's disease; AMPA: alpha-amino-3-hydroxy-5-methyl-4-isoxazolepropionic acid; BDNF: brain-derived neurotrophic factor; i.c.v.: intracerebroventricular; MLK: mixed-lineage kinase; NGF: nerve growth factor; NT: neurotrophin; Trk: tropomyosin-related kinase.

## Competing interests

The authors declare that they have no competing interests.

## Authors' contributions

NJGW tested the NGF mimics and wrote the manuscript and MCP synthesized the NGF mimics.
